# Methyl 2-[5-(4-hydroxy­phen­yl)-3-methyl­sulfanyl-1-benzofuran-2-yl]acetate

**DOI:** 10.1107/S1600536809037763

**Published:** 2009-09-26

**Authors:** Hong Dae Choi, Pil Ja Seo, Byeng Wha Son, Uk Lee

**Affiliations:** a Department of Chemistry, Dongeui University, San 24 Kaya-dong Busanjin-gu, Busan 614-714, Republic of Korea; b Department of Chemistry, Pukyong National University, 599-1 Daeyeon 3-dong, Nam-gu, Busan 608-737, Republic of Korea

## Abstract

In the title compound, C_18_H_16_O_4_S, the 4-hydroxy­phenyl ring is rotated out of the benzofuran plane, making a dihedral angle of 34.52 (6)°. The methyl group of the methyl­sulfanyl substituent is almost perpendicular to the plane of the benzofuran fragment [100.90 (8)°] and is slightly tilted towards it. The crystal structure is stabilized by inter­molecular O—H⋯O hydrogen bonds, and by inter­molecular C—H⋯π inter­actions between a methyl H atom of the methyl­sulfanyl substituent and the 4-hydroxy­phenyl ring.

## Related literature

For the crystal structures of similar alkyl 2-[5-(4-hydroxy­phen­yl)-3-methyl­sulfanyl-1-benzofuran-2-yl]acetate derivatives, see: Choi *et al.* (2006 ▶, 2009 ▶). For the pharmacological activity of benzofuran compounds, see: Howlett *et al.* (1999 ▶); Twyman & Allsop (1999 ▶). For natural products involving a benzofuran ring, see: Akgul & Anil (2003 ▶); von Reuss & König (2004 ▶).
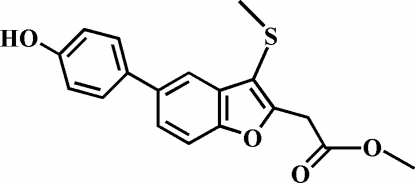


## Experimental

### 

#### Crystal data

C_18_H_16_O_4_S
*M* *_r_* = 328.37
Monoclinic, 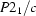

*a* = 13.6661 (6) Å
*b* = 7.6643 (3) Å
*c* = 15.6344 (7) Å
β = 95.4731 (6)°
*V* = 1630.10 (12) Å^3^
*Z* = 4
Mo *K*α radiation
μ = 0.22 mm^−1^
*T* = 173 K
0.45 × 0.25 × 0.25 mm

#### Data collection

Bruker SMART CCD diffractometer
Absorption correction: multi-scan (*SADABS*; Sheldrick, 2000 ▶) *T*_min_ = 0.940, *T*_max_ = 0.961
13917 measured reflections
3703 independent reflections
2894 reflections with *I* > 2σ(*I*)
*R*_int_ = 0.046

#### Refinement

*R*[*F* ^2^ > 2σ(*F* ^2^)] = 0.035
*wR*(*F* ^2^) = 0.097
*S* = 1.08
3703 reflections
213 parameters
H atoms treated by a mixture of independent and constrained refinement
Δρ_max_ = 0.30 e Å^−3^
Δρ_min_ = −0.25 e Å^−3^

### 

Data collection: *SMART* (Bruker, 2001 ▶); cell refinement: *SAINT* (Bruker, 2001 ▶); data reduction: *SAINT*; program(s) used to solve structure: *SHELXS97* (Sheldrick, 2008 ▶); program(s) used to refine structure: *SHELXL97* (Sheldrick, 2008 ▶); molecular graphics: *ORTEP-3* (Farrugia, 1997 ▶) and *DIAMOND* (Brandenburg, 1998 ▶); software used to prepare material for publication: *SHELXL97*.

## Supplementary Material

Crystal structure: contains datablocks global, I. DOI: 10.1107/S1600536809037763/si2201sup1.cif

Structure factors: contains datablocks I. DOI: 10.1107/S1600536809037763/si2201Isup2.hkl

Additional supplementary materials:  crystallographic information; 3D view; checkCIF report

## Figures and Tables

**Table 1:** Hydrogen-bond geometry (Å, °)

*D*—H⋯*A*	*D*—H	H⋯*A*	*D*⋯*A*	*D*—H⋯*A*
O4—H4⋯O3^i^	0.81 (3)	1.97 (3)	2.7826 (18)	177 (2)
C18—H18*C*⋯*Cg*^ii^	0.96	2.88	3.762 (2)	153

Symmetry codes: (i) 
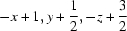
; (ii) 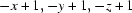. *Cg* is the centroid of the C12–C17 phenyl ring.

**Fig. 1.**
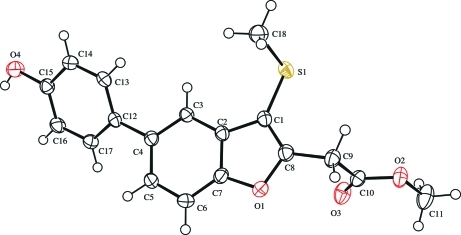
The molecular structure of the title compound with the atom numbering scheme. Displacement ellipsoids are drawn at the 50% probability level. H atoms are presented as a small cycles of arbitrary radius.

**Fig. 2.**
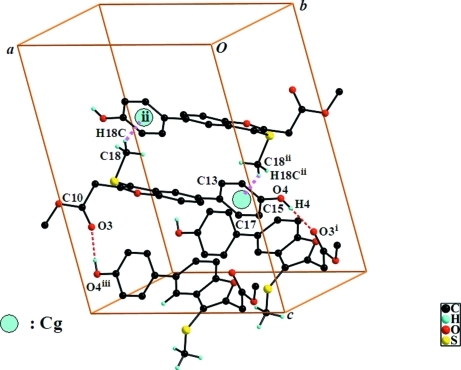
O–H···O and C—H···π interactions (dotted lines) in the title compound. Cg denotes the ring centroid. [Symmetry codes: (i) - x + 1, y + 1, - z + 3/2; (ii) - x + 1, - y + 1, - z + 1; (iii) - x + 1, y - 1/2, - z + 3/2.]

## supplementary crystallographic information

### Comment

Benzofuran ring systems have received considerable interest in view of their pharmacological properties (Howlett *et al.*, 1999; Twyman & Allsop, 1999), and these compounds are occurring in natural products (Akgul & Anil, 2003; von Reuss & König, 2004). As a part of our ongoing studies of the effect of side chain substituents on the solid state structures of alkyl 2-[5-(4-hydroxyphenyl)-3-methylsulfanyl-1-benzofuran-2-yl]acetate analogues (Choi *et al.*, 2006, 2009), we report the crystal structure of the title compound (Fig. 1).

The benzofuran unit is essentially planar, with a mean deviation of 0.005 (1) Å from the least-squares plane defined by the nine constituent atoms. The 4-hydroxyphenyl ring is rotated out of the benzofuran plane, making a dihedral angle of 34.52 (6)°. The methyl group of the methylsulfanyl substituent is tilted towards the plane of the benzofuran unit [100.90 (8)°]. The molecular packing (Fig. 2) is stabilized by intermolecular O—H···O hydrogen bonds between the hydroxy H atom and the oxygen of the C═O unit, with a O4—H4···O3^i^ (Table 1). The crystal packing (Fig. 2) is further stabilized by intermolecular C—H···π interactions between the methyl H atom of the methylsulfanyl substituent and the 4-hydroxyphenyl ring, with a C18—H18C···Cg^ii^ (Table 1; Cg is the centroid of the C12–C17 phenyl ring).

### Experimental

2-[5-(4-Hydroxyphenyl)-3-methylsulfanyl-1-benzofuran-2-yl]acetic acid (471 mg, 1.5 mmol) was added to a solution of concentrated sulfuric acid (3 drops) in methanol (20 ml), and the mixture was refluxed for 6h, then cooled. The solvent was evaporated and the residue was poured into water. The mixture was extracted with dichloromethane, dried over magnesium sulfate, filtered and concentrated under vacuum. The residue was purified by column chromatography (benzene–acetone, 9 : 1 v/v) to afford the title compound as a colorless solid [yield 88%, m.p. 446–447 K; *R*_f_ = 0.49 (benzene–acetone, 9 : 1 v/v)]. Single crystals suitable for X-ray diffraction were prepared by evaporation of a solution of the title compound in benzene at room temperature.

### Refinement

The hydroxy H atom was found in a difference Fourier map and refined freely. The other H atoms were positioned geometrically and refined using a riding model, with C—H = 0.93 Å for the aryl, 0.97 Å for the methylene, and 0.96 Å for the methyl H atoms. *U*_iso_(H) = 1.2*U*_eq_(C) for the aryl and methylene H atoms, and 1.5*U*_eq_(C) for methyl H atoms.

### Crystal data

**Table:**

C_18_H_16_O_4_S	*F*(000) = 688
*M**_r_* = 328.37	*D*_x_ = 1.338 Mg m^−^^3^
Monoclinic, *P*2_1_/*c*	Mo *K*α radiation, λ = 0.71073 Å
Hall symbol: -P 2ybc	Cell parameters from 6226 reflections
*a* = 13.6661 (6) Å	θ = 2.6–27.3°
*b* = 7.6643 (3) Å	µ = 0.22 mm^−^^1^
*c* = 15.6344 (7) Å	*T* = 173 K
β = 95.4731 (6)°	Block, colorless
*V* = 1630.10 (12) Å^3^	0.45 × 0.25 × 0.25 mm
*Z* = 4	

### Data collection

**Table:**

Bruker SMART CCD diffractometer	3703 independent reflections
Radiation source: fine-focus sealed tube	2894 reflections with *I* > 2σ(*I*)
graphite	*R*_int_ = 0.046
Detector resolution: 10.0 pixels mm^-1^	θ_max_ = 27.5°, θ_min_ = 1.5°
φ and ω scans	*h* = −17→17
Absorption correction: multi-scan (*SADABS*; Sheldrick, 2000)	*k* = −9→9
*T*_min_ = 0.940, *T*_max_ = 0.961	*l* = −20→20
13917 measured reflections	

### Refinement

**Table:**

Refinement on *F*^2^	Primary atom site location: structure-invariant direct methods
Least-squares matrix: full	Secondary atom site location: difference Fourier map
*R*[*F*^2^ > 2σ(*F*^2^)] = 0.035	Hydrogen site location: difference Fourier map
*wR*(*F*^2^) = 0.097	H atoms treated by a mixture of independent and constrained refinement
*S* = 1.08	*w* = 1/[σ^2^(*F*_o_^2^) + (0.038*P*)^2^ + 0.664*P*] where *P* = (*F*_o_^2^ + 2*F*_c_^2^)/3
3703 reflections	(Δ/σ)_max_ < 0.001
213 parameters	Δρ_max_ = 0.30 e Å^−^^3^
0 restraints	Δρ_min_ = −0.25 e Å^−^^3^

### Special details

**Table:**

Geometry. All esds (except the esd in the dihedral angle between two l.s. planes) are estimated using the full covariance matrix. The cell esds are taken into account individually in the estimation of esds in distances, angles and torsion angles; correlations between esds in cell parameters are only used when they are defined by crystal symmetry. An approximate (isotropic) treatment of cell esds is used for estimating esds involving l.s. planes.
Refinement. Refinement of F^2^ against ALL reflections. The weighted R-factor wR and goodness of fit S are based on F^2^, conventional R-factors R are based on F, with F set to zero for negative F^2^. The threshold expression of F^2^ > 2sigma(F^2^) is used only for calculating R-factors(gt) etc. and is not relevant to the choice of reflections for refinement. R-factors based on F^2^ are statistically about twice as large as those based on F, and R- factors based on ALL data will be even larger.

### Fractional atomic coordinates and isotropic or equivalent isotropic displacement parameters (Å^2^)

**Table:**

	*x*	*y*	*z*	*U*_iso_*/*U*_eq_	
S1	0.69956 (3)	0.01335 (5)	0.51042 (3)	0.02878 (12)	
O1	0.80580 (8)	0.44720 (15)	0.62041 (7)	0.0290 (3)	
O2	1.05303 (8)	0.08785 (16)	0.60370 (7)	0.0330 (3)	
O3	0.94920 (8)	0.15452 (18)	0.70121 (8)	0.0379 (3)	
O4	0.09318 (9)	0.48769 (18)	0.64861 (9)	0.0377 (3)	
H4	0.0789 (17)	0.535 (3)	0.6922 (17)	0.061 (8)*	
C1	0.72205 (11)	0.2164 (2)	0.55937 (9)	0.0244 (3)	
C2	0.65254 (11)	0.33024 (19)	0.59592 (9)	0.0227 (3)	
C3	0.55110 (11)	0.3275 (2)	0.60033 (9)	0.0229 (3)	
H3	0.5133	0.2362	0.5759	0.027*	
C4	0.50698 (11)	0.4634 (2)	0.64192 (9)	0.0236 (3)	
C5	0.56623 (11)	0.6008 (2)	0.67804 (10)	0.0277 (3)	
H5	0.5365	0.6911	0.7057	0.033*	
C6	0.66711 (12)	0.6063 (2)	0.67373 (10)	0.0291 (3)	
H6	0.7054	0.6977	0.6974	0.035*	
C7	0.70754 (11)	0.4684 (2)	0.63246 (10)	0.0254 (3)	
C8	0.81116 (11)	0.2933 (2)	0.57520 (10)	0.0268 (3)	
C9	0.91070 (11)	0.2406 (2)	0.55381 (10)	0.0289 (3)	
H9A	0.9450	0.3428	0.5354	0.035*	
H9B	0.9040	0.1591	0.5062	0.035*	
C10	0.97139 (11)	0.1573 (2)	0.62834 (10)	0.0266 (3)	
C11	1.11920 (14)	0.0120 (3)	0.67179 (13)	0.0460 (5)	
H11A	1.0857	−0.0787	0.6997	0.055*	
H11B	1.1403	0.1006	0.7129	0.055*	
H11C	1.1754	−0.0362	0.6478	0.055*	
C12	0.39861 (11)	0.46601 (19)	0.64631 (10)	0.0227 (3)	
C13	0.33451 (11)	0.4013 (2)	0.57879 (10)	0.0246 (3)	
H13	0.3604	0.3531	0.5312	0.030*	
C14	0.23358 (12)	0.4071 (2)	0.58084 (10)	0.0272 (3)	
H14	0.1925	0.3618	0.5354	0.033*	
C15	0.19364 (11)	0.4809 (2)	0.65104 (10)	0.0267 (3)	
C16	0.25574 (12)	0.5440 (2)	0.71976 (10)	0.0289 (3)	
H16	0.2296	0.5919	0.7673	0.035*	
C17	0.35686 (12)	0.5352 (2)	0.71715 (10)	0.0273 (3)	
H17	0.3979	0.5765	0.7637	0.033*	
C18	0.62715 (13)	0.0771 (2)	0.41243 (12)	0.0365 (4)	
H18A	0.5645	0.1201	0.4260	0.055*	
H18B	0.6176	−0.0220	0.3750	0.055*	
H18C	0.6610	0.1671	0.3843	0.055*	

### Atomic displacement parameters (Å^2^)

**Table:**

	*U*^11^	*U*^22^	*U*^33^	*U*^12^	*U*^13^	*U*^23^
S1	0.0320 (2)	0.0222 (2)	0.0323 (2)	0.00435 (16)	0.00399 (16)	−0.00155 (16)
O1	0.0219 (5)	0.0333 (6)	0.0313 (6)	0.0002 (5)	0.0002 (4)	−0.0056 (5)
O2	0.0240 (6)	0.0455 (7)	0.0299 (6)	0.0094 (5)	0.0041 (4)	0.0007 (5)
O3	0.0297 (6)	0.0546 (8)	0.0309 (6)	0.0048 (6)	0.0105 (5)	0.0091 (6)
O4	0.0244 (6)	0.0501 (8)	0.0394 (7)	−0.0021 (5)	0.0082 (5)	−0.0150 (6)
C1	0.0250 (8)	0.0242 (7)	0.0241 (7)	0.0036 (6)	0.0028 (6)	0.0005 (6)
C2	0.0246 (7)	0.0218 (7)	0.0218 (7)	0.0030 (6)	0.0023 (6)	0.0008 (6)
C3	0.0238 (7)	0.0210 (7)	0.0239 (7)	0.0001 (6)	0.0020 (6)	−0.0002 (6)
C4	0.0253 (7)	0.0241 (8)	0.0212 (7)	0.0030 (6)	0.0011 (6)	0.0011 (6)
C5	0.0279 (8)	0.0268 (8)	0.0277 (8)	0.0059 (6)	−0.0004 (6)	−0.0059 (6)
C6	0.0299 (8)	0.0275 (8)	0.0289 (8)	−0.0013 (7)	−0.0031 (6)	−0.0064 (6)
C7	0.0210 (7)	0.0300 (8)	0.0246 (8)	0.0020 (6)	−0.0006 (6)	−0.0003 (6)
C8	0.0259 (8)	0.0305 (8)	0.0240 (7)	0.0033 (6)	0.0019 (6)	−0.0001 (6)
C9	0.0227 (8)	0.0370 (9)	0.0274 (8)	0.0022 (7)	0.0039 (6)	0.0001 (7)
C10	0.0204 (7)	0.0295 (8)	0.0304 (8)	−0.0025 (6)	0.0055 (6)	−0.0002 (6)
C11	0.0311 (9)	0.0670 (14)	0.0392 (10)	0.0158 (9)	0.0007 (8)	0.0111 (10)
C12	0.0255 (7)	0.0190 (7)	0.0238 (7)	0.0035 (6)	0.0033 (6)	0.0010 (6)
C13	0.0290 (8)	0.0223 (7)	0.0232 (7)	0.0026 (6)	0.0061 (6)	−0.0021 (6)
C14	0.0279 (8)	0.0276 (8)	0.0257 (8)	−0.0010 (6)	0.0006 (6)	−0.0038 (6)
C15	0.0240 (7)	0.0268 (8)	0.0299 (8)	0.0001 (6)	0.0057 (6)	−0.0007 (6)
C16	0.0311 (8)	0.0314 (8)	0.0252 (8)	0.0018 (7)	0.0080 (6)	−0.0054 (7)
C17	0.0295 (8)	0.0284 (8)	0.0239 (8)	0.0017 (6)	0.0015 (6)	−0.0039 (6)
C18	0.0385 (10)	0.0337 (9)	0.0361 (9)	0.0017 (8)	−0.0020 (7)	−0.0023 (7)

### Geometric parameters (Å, °)

**Table:**

S1—C1	1.7488 (16)	C8—C9	1.487 (2)
S1—C18	1.8099 (18)	C9—C10	1.506 (2)
O1—C8	1.3810 (19)	C9—H9A	0.9700
O1—C7	1.3833 (18)	C9—H9B	0.9700
O2—C10	1.3260 (18)	C11—H11A	0.9600
O2—C11	1.451 (2)	C11—H11B	0.9600
O3—C10	1.2067 (19)	C11—H11C	0.9600
O4—C15	1.3708 (19)	C12—C13	1.397 (2)
O4—H4	0.81 (3)	C12—C17	1.398 (2)
C1—C8	1.354 (2)	C13—C14	1.383 (2)
C1—C2	1.447 (2)	C13—H13	0.9300
C2—C7	1.390 (2)	C14—C15	1.392 (2)
C2—C3	1.395 (2)	C14—H14	0.9300
C3—C4	1.395 (2)	C15—C16	1.391 (2)
C3—H3	0.9300	C16—C17	1.388 (2)
C4—C5	1.413 (2)	C16—H16	0.9300
C4—C12	1.489 (2)	C17—H17	0.9300
C5—C6	1.387 (2)	C18—H18A	0.9600
C5—H5	0.9300	C18—H18B	0.9600
C6—C7	1.380 (2)	C18—H18C	0.9600
C6—H6	0.9300		
			
C1—S1—C18	100.90 (8)	O3—C10—O2	124.16 (15)
C8—O1—C7	105.64 (12)	O3—C10—C9	124.63 (14)
C10—O2—C11	115.50 (13)	O2—C10—C9	111.21 (13)
C15—O4—H4	108.3 (17)	O2—C11—H11A	109.5
C8—C1—C2	106.18 (14)	O2—C11—H11B	109.5
C8—C1—S1	125.76 (12)	H11A—C11—H11B	109.5
C2—C1—S1	127.94 (12)	O2—C11—H11C	109.5
C7—C2—C3	119.49 (14)	H11A—C11—H11C	109.5
C7—C2—C1	105.79 (13)	H11B—C11—H11C	109.5
C3—C2—C1	134.71 (14)	C13—C12—C17	117.36 (14)
C2—C3—C4	119.18 (14)	C13—C12—C4	121.01 (13)
C2—C3—H3	120.4	C17—C12—C4	121.63 (14)
C4—C3—H3	120.4	C14—C13—C12	121.72 (14)
C3—C4—C5	119.09 (14)	C14—C13—H13	119.1
C3—C4—C12	120.49 (14)	C12—C13—H13	119.1
C5—C4—C12	120.41 (13)	C13—C14—C15	119.89 (14)
C6—C5—C4	122.50 (14)	C13—C14—H14	120.1
C6—C5—H5	118.8	C15—C14—H14	120.1
C4—C5—H5	118.8	O4—C15—C16	122.95 (14)
C7—C6—C5	116.33 (15)	O4—C15—C14	117.44 (14)
C7—C6—H6	121.8	C16—C15—C14	119.61 (14)
C5—C6—H6	121.8	C17—C16—C15	119.76 (14)
C6—C7—O1	126.20 (14)	C17—C16—H16	120.1
C6—C7—C2	123.41 (14)	C15—C16—H16	120.1
O1—C7—C2	110.39 (13)	C16—C17—C12	121.63 (15)
C1—C8—O1	111.98 (13)	C16—C17—H17	119.2
C1—C8—C9	131.83 (15)	C12—C17—H17	119.2
O1—C8—C9	116.18 (14)	S1—C18—H18A	109.5
C8—C9—C10	112.89 (13)	S1—C18—H18B	109.5
C8—C9—H9A	109.0	H18A—C18—H18B	109.5
C10—C9—H9A	109.0	S1—C18—H18C	109.5
C8—C9—H9B	109.0	H18A—C18—H18C	109.5
C10—C9—H9B	109.0	H18B—C18—H18C	109.5
H9A—C9—H9B	107.8		
			
C18—S1—C1—C8	−114.97 (15)	S1—C1—C8—C9	3.7 (3)
C18—S1—C1—C2	69.48 (15)	C7—O1—C8—C1	−0.77 (17)
C8—C1—C2—C7	−0.71 (17)	C7—O1—C8—C9	179.94 (13)
S1—C1—C2—C7	175.53 (12)	C1—C8—C9—C10	−99.8 (2)
C8—C1—C2—C3	178.91 (17)	O1—C8—C9—C10	79.33 (18)
S1—C1—C2—C3	−4.8 (3)	C11—O2—C10—O3	−2.6 (2)
C7—C2—C3—C4	−0.4 (2)	C11—O2—C10—C9	176.96 (15)
C1—C2—C3—C4	−179.96 (16)	C8—C9—C10—O3	−10.1 (2)
C2—C3—C4—C5	0.4 (2)	C8—C9—C10—O2	170.26 (14)
C2—C3—C4—C12	178.85 (13)	C3—C4—C12—C13	−34.2 (2)
C3—C4—C5—C6	0.1 (2)	C5—C4—C12—C13	144.26 (15)
C12—C4—C5—C6	−178.41 (14)	C3—C4—C12—C17	146.78 (15)
C4—C5—C6—C7	−0.5 (2)	C5—C4—C12—C17	−34.8 (2)
C5—C6—C7—O1	179.75 (14)	C17—C12—C13—C14	0.8 (2)
C5—C6—C7—C2	0.5 (2)	C4—C12—C13—C14	−178.21 (14)
C8—O1—C7—C6	−179.09 (15)	C12—C13—C14—C15	0.8 (2)
C8—O1—C7—C2	0.28 (17)	C13—C14—C15—O4	178.18 (15)
C3—C2—C7—C6	0.0 (2)	C13—C14—C15—C16	−1.8 (2)
C1—C2—C7—C6	179.65 (15)	O4—C15—C16—C17	−178.96 (16)
C3—C2—C7—O1	−179.43 (13)	C14—C15—C16—C17	1.0 (2)
C1—C2—C7—O1	0.26 (17)	C15—C16—C17—C12	0.8 (3)
C2—C1—C8—O1	0.92 (18)	C13—C12—C17—C16	−1.7 (2)
S1—C1—C8—O1	−175.42 (11)	C4—C12—C17—C16	177.40 (15)
C2—C1—C8—C9	−179.93 (16)		

### Hydrogen-bond geometry (Å, °)

**Table:**

*D*—H···*A*	*D*—H	H···*A*	*D*···*A*	*D*—H···*A*
O4—H4···O3^i^	0.81 (3)	1.97 (3)	2.7826 (18)	177 (2)
C18—H18C···Cg^ii^	0.96	2.88	3.762 (2)	153

Symmetry codes: (i) −*x*+1, *y*+1/2, −*z*+3/2; (ii) −*x*+1, −*y*+1, −*z*+1.
